# Development and Evaluation of Solid Dispersion-Based Sublingual Films of Nisoldipine

**DOI:** 10.3390/ph16111589

**Published:** 2023-11-09

**Authors:** Yahya Alhamhoom, Abhay Sharma, Shivakumar Hagalavadi Nanjappa, Avichal Kumar, Anas Alshishani, Mohammed Muqtader Ahmed, Syeda Ayesha Farhana, Mohamed Rahamathulla

**Affiliations:** 1 Department of Pharmaceutics, College of Pharmacy, King Khalid University, Al Faraa, Abha 62223, Saudi Arabia; ysalhamhoom@kku.edu.sa; 2 Department of Pharmaceutics, KLE College of Pharmacy, Bengaluru 560010, India; abhaysharmabph@gmail.com (A.S.); avichalk0994@gmail.com (A.K.); 3 Faculty of Pharmacy, Zarqa University, Zarqa 13132, Jordan; aalshishani@zu.edu.jo; 4 Department of Pharmaceutics, College of Pharmacy, Prince Sattam Bin Abdul Aziz University, Al Kharj 11942, Saudi Arabia; muqtadernano@psau.edu.sa; 5 Department of Pharmaceutics, Unaizah College of Pharmacy, Qassim University, Unaizah 51911, Saudi Arabia; a.farhana@qu.edu.sa

**Keywords:** sublingual films, solid dispersion, phase solubility, in vitro study, hypertension management, nisoldipine

## Abstract

Nisoldipine (NIS) is a calcium channel blocker that exhibits poor bioavailability (~5%) due to low aqueous solubility and presystemic metabolism in the gut wall. In this context, the present work aimed to develop NIS solid dispersion (NISSD)-based sublingual films using solvent casting technique to improve the dissolution. Phase solubility studies indicated that Soluplus^®^ was the most effective carrier for improving the aqueous solubility of NIS. NISSDs were initially developed using the solvent evaporation method. Fourier transform infrared spectrometric studies were found to display the characteristic vibrational bands related to C=O stretching and N-H deformation in NISSDs, proving the chemical integrity of the drug in NISSDs. Subsequently, bioadhesive sublingual films of NISSDs were formulated using solvent casting method, using hydroxypropyl methyl cellulose (HPMC) E5, E15, and hydroxy ethyl cellulose (HEC EF) as hydrophilic polymers and polyethylene glycol 400 (PEG 400) as plasticizer. The incorporation of NISSDs was found to produce clear films that displayed uniform content. The sublingual film of NISSDs composed of HPMC E5 (2% *w*/*v*), was found to display the least thickness (0.29 ± 0.02 mm), the highest folding endurance (168.66 ± 4.50 times), and good bioadhesion strength (12.73 ± 0.503 g/cm^2^). This film was found to rapidly disintegrate (28.66 ± 3.05 sec) and display near-complete drug release (94.24 ± 1.22) in 30 min. Incorporating NISSDs into rapidly bioadhesive sublingual films considerably improves drug dissolution. Overall, these research outcomes underscored the potential of rapidly dissolving bioadhesive sublingual films to evade gut metabolism and resolve the bioavailability issues associated with oral administration of NIS.

## 1. Introduction

Inadequate management of hypertension leads to cardiovascular problems, which are a leading cause of mortality globally. Irrespective of a country’s geographic location or economic standing, hypertension is common everywhere in the world [1]. It is estimated that 47% of the United States’ patients and 55% of European patients are taking antihypertensive medication [2].

According to a recent study, maintaining normal blood pressure has become essential in order to reduce the risk of serious disease and mortality [3,4]. Patients who suffer from acute angina attacks and an immediate increase in blood pressure are more restless and have reduced functional ability [5]. The apparent choice is the administration of anti-hypertensives via the parenteral route, which, however, is invasive and demands medical intervention [3]. Moreover, accessibility to critical care medical centers would be a big challenge for patients in remote, poor clinical settings. Considering this, there is an urgent need to develop a novel pain-free drug delivery system that would be able to elicit a quicker therapeutic response. In this context, the sublingual route, owing to the high vasculature and thin mucosal epithelium (190 µm), has proven to be an attractive option to elicit a faster onset and thus offers promising clinical benefit in the delivery of antianginals in cardiac emergencies [6]. It has to be noted that the thickness of the sublingual mucosa is much thinner (190 µm) compared to other regions of the oral cavity (500–800 µm) [7]. Moreover, the sublingual route of administration is suitable for the elderly and those who have difficulties swallowing (dysphagia), as it provides a precise dose, enables easy administration, elicits a quicker onset of action, and therefore would be a good alternative to liquid oral dosage forms [8].

Nisoldipine (NIS) is a dihydropyridine derivative that acts as a calcium channel blocker. NIS lowers vascular resistance and blood pressure by preventing the uptake of calcium by the myocardium and smooth muscle cells, and therefore is commonly used in the treatment of high blood pressure and angina pectoris [9]. NIS belongs to the BCS Class II as it exhibits poor aqueous solubility (5.77 × 10^−3^ mg/mL at 25 °C and ~5 mg/mL at 37 °C), which results in poor dissolution [10,11]. The drug exhibits poor oral bioavailability (~5%) due low aqueous solubility and extensive presystemic metabolism by CYP3A4 enzymes in the gut wall [12]. This study is based on the on the strong rationale that drugs delivered sublingually can evade the gut metabolism and have improve bioavailability [13]. A drug released from the rapidly dissolving film is primarily absorbed by the sublingual region. Moreover, the film is likely to enhance bioavailability by exposing a pulsed dose of NIS to the CYP3A enzymes in case the drug dissipates into the gastrointestinal tract [14]. The pulsed dose of NIS is likely to saturate the metabolizing enzymes in the gut wall and enhance the bioavailability of drugs prone to presystemic clearance [15]. To the best of our knowledge, there are no reports so far on the development of instantly disintegrating sublingual films of NIS with the aim to elicit a quicker onset and to eventually improve the bioavailability. Amorphous solid dispersions have a proven ability to improve the solubility, dissolution, oral absorption, and eventually the bioavailability of poorly water-soluble drugs [16]. Considering these limitations of NIS, the present study aims to prepare solid dispersions of NIS (NISSDs) with varying percentages of carriers. The optimized NISSD batch will be incorporated into an instantly dispersible film in order to improve dissolution and generate a pulsed dose of NIS at the sublingual region of the oral cavity. The dispersible films will be further assessed for drug–excipient interaction using FTIR, DSC, and X-ray diffraction studies. The evaluation of the general appearance, weight variations, thickness, drug content, folding endurance, surface pH, disintegration time, in vitro drug release and ex vivo mucoadhesion of the films are a part of the present investigation.

## 2. Results

### 2.1. Phase Solubility Studies of the Carrier

**Figure 1:**
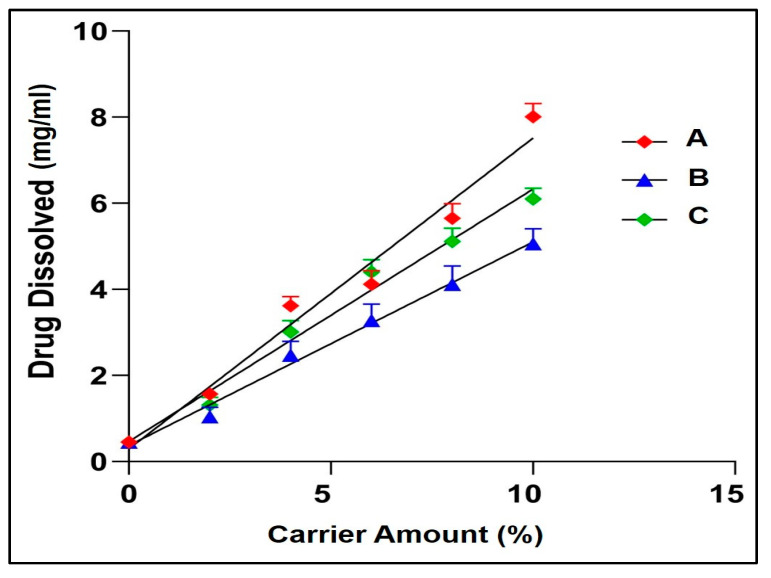
Phase solubility profile at different concentrations of carriers (**A**) Soluplus^®^, (**B**) Kolliphor RH-40, (**C**) PVP K30.

NIS has a solubility of 0.25 mg/mL in water at room temperature. The phase solubility studies indicated that the solubility of NIS was found to increase linearly with an increase in the concentration of the carriers used. Soluplus^®^ was found to increase the solubility of NIS significantly (*p* < 0.001) to 8.01 ± 0.31 mg/mL when compared to PVP K30 (6.10 ± 0.25 mg/mL) or Kolliphor RH40 (5.06 ± 0.35 mg/mL) at a carrier concentration of 10%. The effect of Soluplus^®^ on enhancing the aqueous solubility of NIS is clearly evident in Table 1 and Figure 1.

### 2.2. Evaluation of Sublingual Films

#### 2.2.1. Bright Field Microscopy

**Figure 2:**
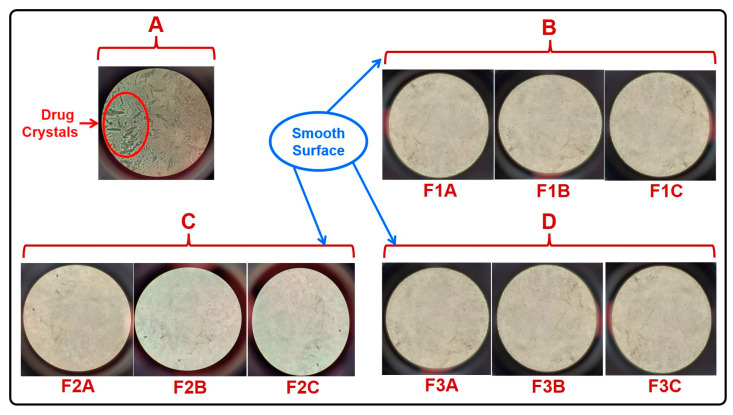
Bright-field microscopic images of (**A**) a film with the pure drug, (**B**–**D**) all film formulations with solid dispersions.

The morphology and surface topography observed using a bright-field microscope revealed that films composed of NIS alone indicated the presence of drug crystals (Figure 2A). On the contrary, the rapidly dispersible films of the batches F1A, F1B, F1C, F2A, F2B, F2C, F3A, F3B, and F3C composed of NISSDs were found to have smooth surfaces with no evidence of drug crystals as indicated by bright-field microscopy images (Figure 2B–D).

#### 2.2.2. Compatibility Studies

**Figure 3:**
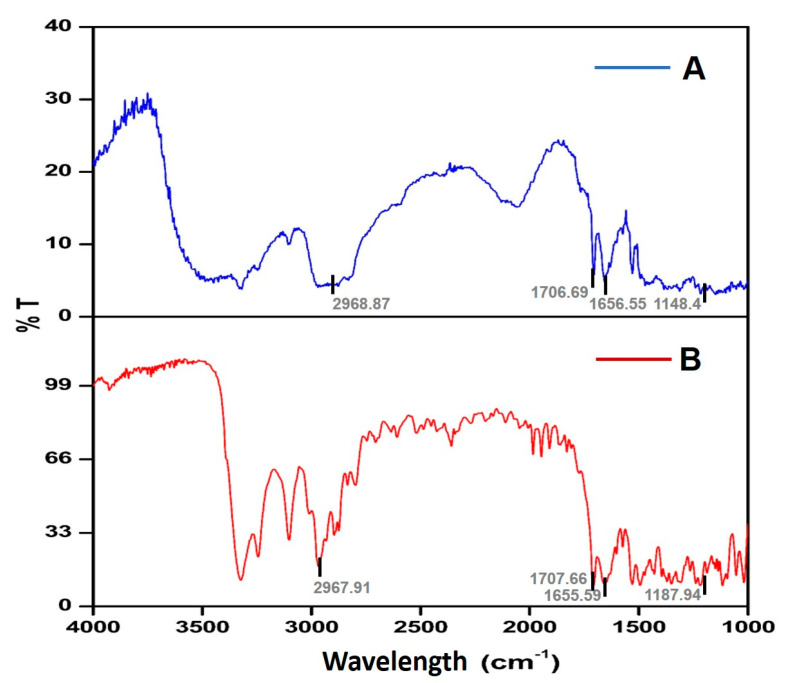
FTIR vibrational spectra of F2A (**A**) and nisoldipine (**B**).

The FTIR vibrational spectroscopy of NIS displayed characteristic vibrational bands at 2967.91, 1707.66, 1655.59, and 1187.94 cm^−1^. The FTIR vibrational spectra of the NISSD formulations and their physical mixtures also revealed the characteristic NIS peaks in the same area as shown in Figure 3 and Table 2.

#### 2.2.3. Weight Variation

The average weight of the films in different batches ranged from 25.67 ± 2.82 mg to 75.15 ± 3.39 mg. The uniformity in weight was a good indication of the reproducibility of the film casting technique.

#### 2.2.4. Thickness Measurement

**Figure 4:**
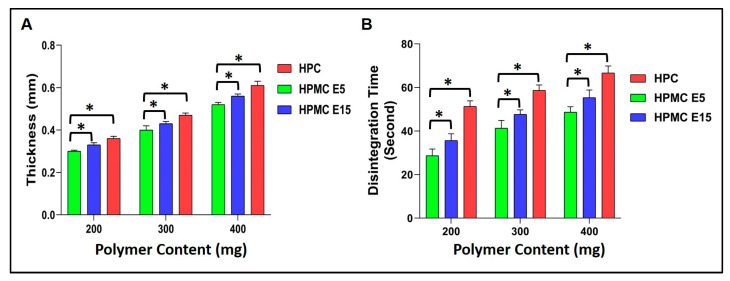
Thickness (**A**) and disintegration time (**B**) of films casted with varying amounts of polymer (* showing level of significance, *p* < 0.05).

The thickness average of each formulated sublingual film of different batches ranged from 0.29 ± 0.005 mm to 0.61 ± 0.025 mm, as indicated in Figure 4A. The uniformity in thickness indicated the reproducibility of the film casting technique.

#### 2.2.5. Drug Content

Sublingual formulations from distinct batches ranged in drug content from 71.57 ± 2.76% to 96.79 ± 0.63%. The assay values for different batches of films are illustrated in Table 3. The assay values were found to differ from polymer to polymer. The films composed of HPMC E5 were found to display uniform drug content.

#### 2.2.6. Folding Endurance

The values of the folding endurance for different film formulations were found to vary between 92.66 ± 5.03 times and 173.33 ± 4.5 times. The strong folding endurance values revealed the good flexibility and mechanical strength of the films formulated using NISSDs.

#### 2.2.7. Surface pH

The pH values for all formulations ranged from 6.26 ± 0.057 to 6.66 ± 0.057, as portrayed in Table 3. Thus, the films formulated displayed a surface pH that was close to neutral and the physiological pH.

#### 2.2.8. Tack Test

The formulations were all found to be non-tacky, as illustrated in Table 3. The films’ lack of sticky texture indicates they were dry.

#### 2.2.9. Differential Scanning Calorimetry

**Figure 5:**
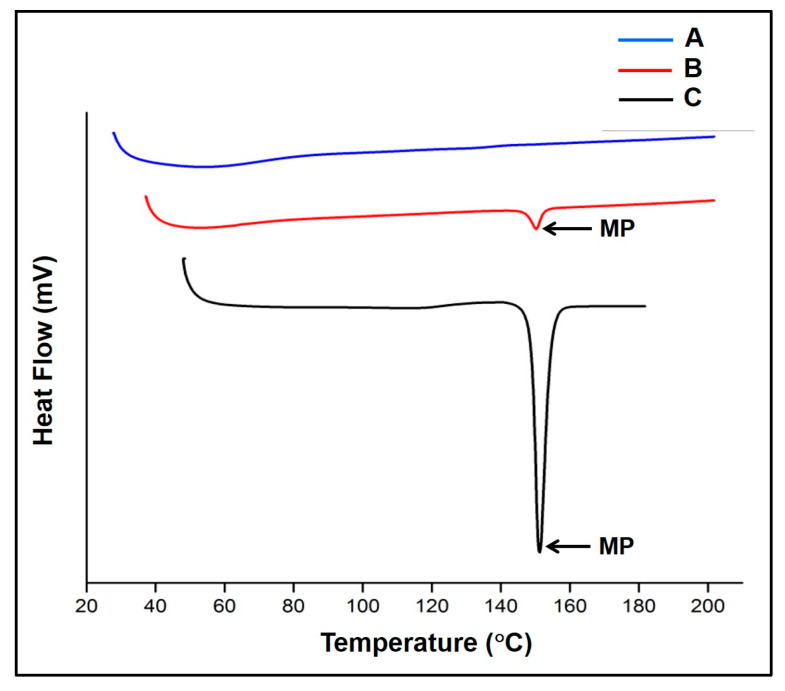
Differential scanning calorimetry report of (**A**) F2A, (**B**) physical mixture, and (**C**) nisoldipine. (MP—melting point).

The DSC thermogram of NIS displayed a sharp endothermic peak at 151.74 °C that corresponded to the melting point of NIS. The thermogram of the physical mixture displayed the low-intensity drug characteristic peak at 150.11 °C, indicating that the drug was rendered nearly amorphous in the physical mixture. However, the thermogram of the NISSD formulation F2A displayed no such drug characteristic peaks indicating its amorphous state as shown in Figure 5 and Table 4.

#### 2.2.10. X-ray Diffraction

**Figure 6:**
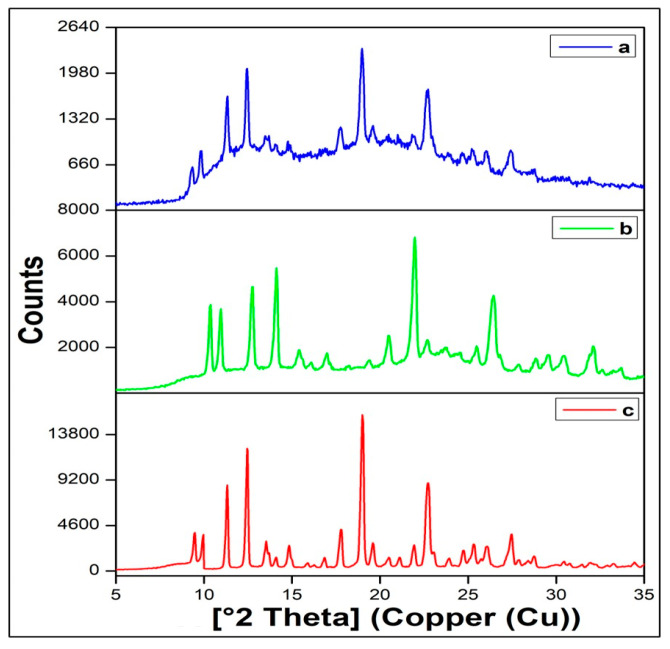
X-ray diffraction report of (**a**) F2A, (**b**) a physical mixture, and (**c**) nisoldipine.

The X-ray diffractogram of NIS was found to display a total of 10 peaks between 10 and 30 2θ values. The physical mixture was found to exhibit the same number of low-intensity characteristic peaks in the same 2θ range indicating its nearly amorphous nature. On the other hand, the formulation F2A displayed a very low-intensity drug characteristic peak in the X-ray diffractogram (Figure 6). The diffractograms indicate that the drug was rendered amorphous as it represented a small fraction of the mixture. The degree of crystallinity was found to drop to nearly 15% for the formulation F2A.

#### 2.2.11. Disintegration Time

Nisoldipine sublingual films were found to disintegrate very quickly. The type and quantity of polymer utilized significantly affected the disintegration time. In salivary phosphate buffer pH 6.8, the disintegration time was observed to vary from 28.66 ± 3.05 to 66.66 ± 3.21 sec, as shown in Figure 4B.

#### 2.2.12. In Vitro Dissolution Studies

**Figure 7:**
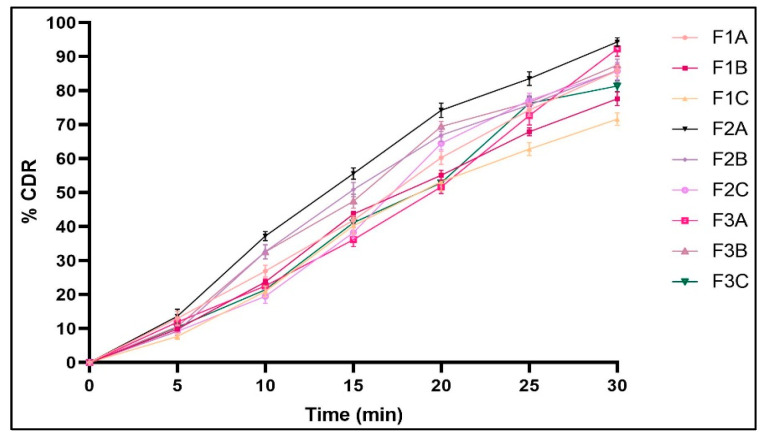
In vitro release profile of nisoldipine from prepared sublingual films.

The dissolution of the sublingual films was expressed as a percentage of the cumulative drug release at various time points. The drug release for various batches of sublingual film formulations was found to vary between 71.57 ± 1.84% and 94.24 ± 1.22% in pH 6.8 phosphate buffer after 30 min, as depicted in Figure 7 and Table 3.

#### 2.2.13. Ex Vivo Mucoadhesion Strength

The bioadhesive films were found to exhibit good mucoadhesion to the buccal mucosa used as a substrate. The observed mucoadhesion strength was found to be 12.73 ± 0.503 gm/cm^2^ for batch F2A.

## 3. Discussion

The present work aimed to improve the bioavailability of NIS by fabricating a rapidly disintegrating sublingual NISSD film to evade the extensive metabolism in the gut wall. Phase solubility was carried out employing a polyvinyl caprolactam–polyvinyl acetate–polyethylene glycol graft copolymer (Soluplus^®^), polyvinylpyrrolidone k25 (PVP k25), and glycerol polyethylene glycol hydroxyl-stearate (Kolliphor RH40). The apparent solubility of NIS in water was determined to be 0.25 mg/mL, as shown in Table 1. The observed value was in close agreement with that reported in the literature [17]. It was found that the NIS’s solubility increased linearly as the carrier concentration increased in all cases. The phase solubility diagram may be classified as AL type as the apparent solubility of NIS increased linearly with carrier concentrations. Soluplus^®^ was found to display a significantly higher (*p* < 0.001) slope compared to Kolliphor RH40 and PVP k25, indicating its ability to improve drug solubility compared to other carriers. Soluplus^®^ is a novel solubilizer having an amphiphilic molecular structure with an HLB value of ~14. The large number of hydroxyl groups facilitates micellar solubilization by molecular interaction. Additionally, Soluplus^®^ dissolves to form micellar structures at concentrations exceeding 7.6 mg/L, which represents the critical micellar concentration (CMC) [18]. The slope value of less than unity indicated the formation of solid dispersion in a stoichiometric ratio of 1:1. Considering that, Soluplus^®^ was used to produce solid dispersions that were eventually loaded into the sublingual films.

Different bioadhesive hydrophilic polymers, such as HPC EF Pharm, HPMC E5, and HPMC E15, were used to formulate NIS sublingual films employing the solvent casting process [19,20]. For the formulation of films, low-crosslinking-density hydrophilic polymers with low viscosity grades were typically used, as they would quickly disintegrate to release the drug [21].

All the film formulations containing NISSDs developed using Soluplus^®^ as a carrier and PEG 400 as a plasticizer were found to be smooth, clear, and non-tacky compared to those fabricated employing the drug as such. However, crystals were observed in the films fabricated with the drug alone, as portrayed in the bright-field microscopic images. The evaporation of ethanol would be the likely reason for the deposition of crystals in the fabricated films [22].

FTIR vibrational spectroscopy studies indicated that NIS showed prominent vibrational bands at 2698, 1707, 1655, and 1188 cm^−1^. The band at 1707 cm^−1^ may be assigned to C=O stretching, while the band at 2697 cm^−1^ is probably caused by N-H deformation [23]. No chemical interaction was found to be likely between the drug and the excipients used, since all of the prominent peaks of NIS were present in the formulation too. The spectral observations demonstrated the chemical integrity of the drug in the formulation and also ruled out any chances of drug and excipient interactions.

Based on the type and quantity of polymer used for the films’ formulation, the thickness of the films was found to differ, as depicted in Table 5. The thickness was generally found to increase with an increase in the amount of polymers incorporated. The thickness of HPMC E5 was found to be significantly lower (*p* < 0.05) compared to other polymers used in the same amounts. The film formed by HPMC E5 exhibited the least thickness, ranging from 0.29 ± 0.02 mm to 0.52 ± 0.01 mm, compared to those fabricated using HPMC E15, which displayed thicknesses varying from 0.31 ± 0.02 mm to 0.56 ± 0.01 mm. The film coated with HPC EF displayed the highest thickness, which ranged from 0.36 ± 0.01 to 0.61 ± 0.02 mm.

The assay value of sublingual films was found to be in the range of 71.57 ± 2.76% to 96.79 ± 0.63%. Films casted using HPMC E5 and HPMC E15 showed a more consistent distribution of the drug, whereas films casted employing HPC EF displayed an uneven distribution of the drug. It was noted that the nature and amount of polymer determine the viscosity, which in turn determines the content uniformity. It is to be noted that HPMC E5 has a lower molecular weight (28,700 Da), followed by HPMC E15 (60,000 Da), and finally HPC (80,000 Da) [24]. The casting solutions that were less viscous were found to demonstrate better content uniformity.

The folding endurance studies indicated that the films fabricated were elastic and had good mechanical properties. The good physicomechanical properties of the films justified the selection of the ideal polymers for the fabrication of the sublingual films. The plasticizer employed, too, would affect the folding endurance. Generally, it was noted that lower amounts of plasticizer rendered the film brittle, while higher amounts resulted in films that were tacky. In the formulation of orodispersible films, polyethylene glycol was utilized as a plasticizer in a proportion equivalent to the dry weight of the polymer [25].

The surface pH is a crucial factor in the assessment of orodispersible films because extreme surface pH ranges are likely to irritate the oral mucosa. In order to minimize the risk of irritation potential, the surface of the sublingual film ideally must be close to neutral [26]. The sublingual films produced in this study are unlikely to irritate the mucosa since their surface pH values are relatively close to neutral.

The tack test indicated that all of the patches were non-tacky, which is likely to lead to quick disintegration of the film in the sublingual region. More importantly, the quantity of the hydrophilic polymer utilized to formulate the sublingual films and the molecular weight of the polymer were shown to determine the disintegration time. Good film-forming properties are attained when HPMC E5 is blended with plasticizers like glycols [27].

DSC thermograms for NIS, physical mixture, and the formulation F2A are captured in Figure 5. The enthalpy of fusion (∆Hf) value of −160.82 J/g and sharp peak of NIS at 151.24 °C confirm the drug’s solid state crystalline form. The observation is consistent with NIS’s melting point, which is listed as 151.29 °C [28]. A sharp endothermic peak characteristic of NIS, indicating the drug’s crystallinity, was found to appear in the physical mixture at 150.11 °C at low intensity, indicating the prevalence of drug crystallinity in the physical mixture as well. However, the complete absence of the drug peak in the formulation F2A indicates the complete amorphization of NIS, which would likely form a solid–solid solution in the polymers. The degree of crystallinity was found to decrease to 11.15% in the physical mixture, while it further dropped below 1% in the film formulation. A reduction in the enthalpy of fusion is indicative of formation of an amorphous solid dispersion [29].

The drug’s solid state in the sublingual film (F2A) was further confirmed using XRD. The X-ray diffractograms were found to be in agreement with the findings in the DSC report. Figure 6 displays the XRD spectra of the NIS, physical mixture, and sublingual film. NIS was found to display eight sharp peaks at 8.5°, 9.2°, 10°, 11.9°, 12.5°, 17.3°, 20°, and 28.1° 2θ value. The same number of peaks was found to appear nearly in the same range in the X-ray diffractogram of the physical mixture, indicating the retention of the drug’s crystallinity. However, the drug was rendered more amorphous as crystallinity dropped to 44% in the physical mixture and to 15% in the film formulation, as per the XRD spectral studies. Thus, the results of DSC and PXRD collectively demonstrated that the NIS is most likely present as a solid–solid solution that would likely to improve the drug dissolution. Moreover, the decrease in the peak intensity can also be attributed to the high excipient amounts present in the formulations [30].

Ex vivo mucoadhesion studies indicated that the films were found to display just sufficient bioadhesion to remain at the application site until disintegration. Generally, the mucoadhesion of a polymer depends on its hydrophilicity, the functional groups, the molecular weight, the extent of crosslinking, and the concentration. The interpenetration of polymer molecules is favored by low-molecular-weight polymers, while entanglement is observed with high-molecular-weight polymers. The optimum molecular weight for maximum mucoadhesion depends on the nature of the polymer, and the adhesive force is found to increase with the molecular weight of the polymer up to 100,000 [31]. HPMC is reported to be tough, elastic, and bioadhesive in vivo, and it is a good choice to fabricate mouth-dissolving films [32].

Further, it was observed that the quantity of the hydrophilic polymer utilized to formulate the sublingual films and the molecular weight of the polymer were found to affect the disintegration time. The films formed with lower amounts of polymers were found to disintegrate quickly as they were thinner. Likewise, faster disintegration was observed with low-molecular-weight polymers, indicating a direct relationship between the disintegration time and the molecular weight of the hydrophilic polymer. In the present study, films fabricated with HPMC E5 displayed a significantly lower disintegration time (*p* < 0.05) compared to films produced using other polymers. Generally, films fabricated with low-molecular-weight polymers are likely to result in quicker disintegration. Thin films produced with a lower amount of polymers invariably have a shorter disintegration time, as per the earlier reports as well [33].

The dissolution profiles of different formulations of sublingual films are illustrated in Figure 7. The amount dissolved was found to depend on the disintegration time. The disintegration time appeared to be the rate-limiting step, as it was found to determine the amount of drug released in 30 min. In addition, the nature of the polymer incorporated affected the drug release. The films formed with HPMC E5 displayed a drug release of 94.24 ± 1.22% after 30 min, which was found to be significantly higher (*p* < 0.05) compared to those fabricated with other polymers. This was followed by films made of HPMC E15, while films made of HPC EF portrayed the lowest amount of release in the given time, emphasizing that the type and amount of polymer had a considerable impact on the drug’s release. Additionally, the amorphous nature of NIS in NISSDs would explain the quicker release of the films.

## 4. Materials and Methods

### 4.1. Materials

Nisoldipine (NIS) was procured from Lusochimica (S.P.A.), Italy. Hydroxypropyl cellulose (HPC EF Pharm) was a gifted sample from Ashland (I) Pvt. ltd. Hydroxypropyl methyl cellulose (HPMC E5, HPMC E15) and polyethylene glycol (PEG 400) were purchased from S.D. Fine chemicals, Mumbai. Polyvinyl pyrrolidone k-25 was obtained from Loba chemie, Mumbai. Polyvinyl caprolactam–polyvinyl acetate–polyethylene glycol graft copolymer (Soluplus^®^) and Polyoxyl 40 castor oil (Kolliphor^®^ RH40) were generous gifts from BASF Corporation, India.

### 4.2. Phase Solubility Studies

Phase solubility studies were performed at room temperature in triplicate, utilizing a method proposed by Higuchi and Connors [34]. NIS was added in excess amount to separate vials, each containing different concentrations (0–10% *w*/*v*) of different polymers (Soluplus^®^, PVP k30, and Kolliphor^®^ RH40) dissolved in distilled water. The vials were sealed and shaken for 72 h at 37 °C in a thermostatically controlled orbital shaker incubator. Subsequently, samples were centrifuged for 10 min at 3000 rpm. The supernatant was then suitably diluted, and the assay was performed using a UV-visible double-beam spectrophotometer at 238 nm [35]. The solubility of NIS on the Y axis was plotted against the concentration of the carriers on the X axis to obtain the phase solubility plots. The slope and intercept of the phase solubility plots were used to calculate the association constant [36].

### 4.3. Preparation of Solid Dispersion

Solid dispersions of NIS with the selected carrier were prepared using a solvent evaporation method. The polymer that displayed the highest slope in the phase solubility studies was used to prepare solid dispersions by co-dissolving NIS and the carrier in a 1:1 ratio in ethanol. The polymers were initially dissolved in the ethanol (10 mL) until a clear solution was obtained. NIS was then added to the solution with continuous stirring for 45 min. The solution was equilibrated at room temperature until the solvent had completely evaporated. The finished product was finally pulverized and used further to produce the sublingual films [37].

### 4.4. Preparation of Sublingual Films

Sublingual films of NISSDs were formulated employing a solvent casting method [38]. Polymeric solutions were prepared by dissolving the required quantities of polymers, including HPMC (E5 and E15) and hydroxyl propyl cellulose (HPC EF), in ethanol on a magnetic stirrer until they were completely dissolved. Later, NISSDs were added to the above solutions and stirred for 30 min before adding PEG 400 as a plasticizer. The solutions were then sonicated to remove any bubbles before being cast into lubricated petri dishes measuring ~9 cm^2^ in area. The Petri dishes were left under an inverted funnel and left to dry for 72 h at room temperature, away from sunlight. The composition of the casting solutions is portrayed in Table 5 [39]. The formed films were then carefully removed from the petri dish and checked visually for any imperfections. The films were then stored in an airtight, amber-colored glass container until further analysis.

### 4.5. Evaluation of Sublingual Films

#### 4.5.1. Bright-Field Microscopy

Bright-field microscopy, a kind of light microscopy that illuminates a sample with visible light, was used to assess the morphology and surface topography of the formulated sublingual films [40]. The formulated films were mounted on a slide and examined under a microscope that has an objective lens and a bright-field condenser (Labomed Vision 2000). Photographs of the samples at appropriate magnifications were acquired.

#### 4.5.2. Compatibility Studies

Drug polymer compatibility studies were assessed using Fourier transform infrared spectrometry (FTIR). The samples were physically mixed with KBr powder, loaded into a diffuse reflectance sampler, and exposed to IR radiation [41]. The data were acquired in the scanning range of 1000–4000 cm^−1^ in a Jasco 450 Plus spectrophotometer to check the chemical integrity of characteristic functional groups of NIS [42].

#### 4.5.3. Weight Variation

Weight variation was evaluated by taking approximately 1 × 1 cm^2^ pieces from three different parts of the casted films and weighing them on an analytical electronic balance (Analytical Balance, Shimadzu BL-220H, Kyoto, Japan). The average weight of each film was recorded, and the results are represented as the mean (±S.D.) of three replicates.

#### 4.5.4. Thickness Measurements

The thickness of the samples was measured using a digital vernier caliper (Mitutoyo Corporation, Tokyo, Japan) with a minimum count of 0.01 mm. Three different points on the film were used to measure the thickness, and the results are expressed as the mean ± S.D. of three replicates.

#### 4.5.5. Drug Content Uniformity

The content of NIS in the film was determined by dissolving a film with a surface area of 1 × 1 cm^2^ in ethanol in a volumetric flask. Later, the resulting solution was suitably diluted with pH 6.8 phosphate buffer. The absorbance of the resulting solution was measured at 237 nm using a visible UV spectrophotometer (Shimadzu UV-1900i spectrophotometer) to determine the drug concentration from the standard calibration curve constructed in ethanol. The evaluation was performed in triplicate, and the findings are expressed as mean ± S.D.

#### 4.5.6. Folding Endurance

The number of folds required to crack or break the film determines the folding endurance; it also determines the brittleness of the film [43]. The folding endurance was measured manually by folding the formulation repeatedly in the same place until it broke. The results are expressed as means (± S.D.) of three determinations [44].

#### 4.5.7. Surface pH Measurement

To check for the potential safety of the film in vivo, the pH of the films’ surface was measured. Using a digital pH meter (Lab Junction, LJ-131), the surface pH was measured. A petri dish containing a test film was filled with 0.5 mL of distilled water and allowed to soak for 30 s. After one minute of equilibration, the electrode of the pH meter was brought into contact with the surface of the film to record the pH. The results are expressed as the mean ± S.D. of three determinations [45].

#### 4.5.8. Tack Test

The tack test, often known as a dryness test, refers to how firmly a film sticks to a biological substrate. Tackiness may also be seen in films that are completely dry [46]. The tackiness of the film is often assessed by physically exerting pressure with the thumb [47].

#### 4.5.9. Differential Scanning Calorimetry

DSC is a thermoanalytical tool employed for identifying a drug’s solid state [48]. The data were acquired for formulated films, physical mixtures, and NIS using a differential scanning calorimeter (DSC-60-Shimadzu, Japan). The samples were taken in aluminum pans and heated at a rate of 10 °C/min while being exposed to a nitrogen atmosphere flowing at a rate of 100 mL/min. Data acquisition was performed in the temperature range of 100 to 200 °C taking into account the melting point of NIS [49]. The degree of crystallinity (Xc) of physical mixtures and formulations relative to NIS was computed using Equation (1).

(1) $$\mathrm{Xc}=\frac{\Delta \mathrm{Hm}\;}{\left(1-w\right).\Delta H{}^{\circ}m\;}\times 100$$

where ∆Hm is the measured heat of the fusion of the sample, ∆H°m is the heat of the fusion of 100% crystalline NIS (Jg^−1^), and W is the weight fraction of polymer and carrier [50].

#### 4.5.10. X-ray Diffraction

The X-ray diffraction studies were carried out in powder using an X-ray diffractometer (LabX XRD-6100). Data on X-ray powder diffraction (XRPD) were collected with a scanning speed of 0.5 sec per step using an apparatus fitted with a Cu radiation source operating at 40 kV and 40 mV. The diffractogram was recorded for the formulated films, physical mixture, and NIS at 40 kV and 40 mV [51]. The percentage crystallinity was calculated according to Equation (2) [52].

(2) $$\mathrm{DC}=\frac{I\;\mathrm{sam}\;}{I\;\mathrm{ref}\;}\times 100$$

where I_sam_ is the highest characteristic peak of the sample and I_ref_ is the peak height at the same angle at the reference with the height intensity.

#### 4.5.11. Disintegration Test

The release of NIS from the formulated films depends on the disintegration time of the film. The disintegration test was performed by placing a (2 × 2 cm^2^) piece of the film in a United States Pharmacopoeia disintegration test (Electrolab ED-2L) apparatus filled with 900 mL of phosphate buffer of pH 6.8. The time taken by the film to completely disintegrate was reported. Every formulation was subjected to six determinations, and the findings are presented as the mean ± S.D. of three determinations [53].

#### 4.5.12. In Vitro Dissolution Studies

The in vitro dissolution study was performed using a United States Pharmacopoeia dissolution tester type II (Electrolab TDT 08T) employing the paddle-over-disc method. The dissolution test was performed with 500 mL of pH 6.8 phosphate buffer as the medium, which was kept at 37 ± 0.5 °C [54]. The film (2 × 2 cm^2^) was glued to the disc with acrylate adhesive, and the paddle was rotated at 50 revolutions per minute above it. At 5, 10, 15, 20, 25, and 30 min, about 5 mL of the sample was withdrawn and replaced with an equal amount of buffer that was kept at the same temperature. The amount of NIS dissolved at various times was determined by measuring the absorbance at 237 nm using a UV spectrophotometer. A graph of the amount of drug release was calculated and plotted against time to determine the dissolution profiles. By comparing solid dispersion dissolution data with formulation dissolution data, the f1 and f2 factors were computed [55].

#### 4.5.13. Ex Vivo Mucoadhesion Strength

The mucoadhesive strength of the formulated film was evaluated using a modified two-arm balance technique with goat buccal mucosa as a substrate [56]. Goat buccal mucosa, having a thickness of 2 mm, was treated with saline buffer and secured to a horizontal surface using glue. The sublingual patch (1 × 1 cm^2^) was secured to the left-hand side pan of the balance and brought into contact with the mucosa for a span of 2 min. At the same position, the balance was brought to equilibrium, and then force was applied by gradually sliding the weights until the patch detached from the mucosal surface. The mucoadhesive strength was quantified in terms of grams.

#### 4.5.14. Statistical Analysis

The results obtained were statistically analyzed using an unpaired Student’s t test in GraphPad Prism version 7. A p value of less than 0.05 was considered to be statistically significant. The data generated are expressed as mean ± standard deviation.

## 5. Conclusions

The solvent casting process was successfully used to produce rapidly dissolving sublingual films of NIS solid dispersions with the most desired features, using HPMC E5 as the film form and PEG400 as the plasticizer. Solid dispersion with Soluplus^®^ was found to improve the aqueous solubility of NIS compared to other carriers. The sublingual films of NISSDs were found to be clear, smooth, and non-tacky. The sublingual films developed using HPMC E5 displayed quick disintegration and rapid dissolution. The sublingual films of NIS are likely to overcome the presystemic gut wall metabolism and therefore improve the bioavailability of NIS. However, the promising results observed warrant further investigations to elucidate the drug pharmacokinetics following sublingual administration in order to titrate the dose. The novel sublingual films, by virtue of the ability to generate a pulsed dose at the absorption site, are likely to overcome the presystemic clearance of several therapeutic agents prone to gut metabolism and improve the bioavailability. The sublingual films would also be the preferred platform for management of hypertension in the geriatric population prone to swallowing difficulties.

## Figures and Tables

**Table 1:** Phase solubility of nisoldipine with different carriers.

% of Carrier	0	2	4	6	8	10
Nisoldipine Dissolved (mg/mL), Mean ± SD						
Soluplus^®^	0.25 ± 0.02	1.57 ± 0.14	3.62 ± 0.21	4.12 ± 0.31	5.65 ± 0.34	8.01 ± 0.31
Kolliphor RH40	0.25 ± 0.02	1.04 ± 0.22	2.47 ± 0.32	3.28 ± 0.38	4.12 ± 0.42	5.06 ± 0.35
PVP K30	0.25 ± 0.02	1.31 ± 0.18	3.01 ± 0.26	4.40 ± 0.29	5.11 ± 0.31	6.10 ± 0.25

**Table 2:** Fourier transform infrared characteristic vibrational bands of nisoldipine and F2A.

Characteristic Vibrational Bands (cm^−1^)	Functional Groups	Band Positions Observed in Nisoldipine (cm^−1^)	Band Positions Observed in Formulation (cm^−1^)
3000–2800	N-H (Stretching)	2967.91	2968.87
1725–1705	C=O (Stretching)	1707.66	1706.69
1662–1626	C=C (Stretching)	1655.59	1656.55
1205–1124	C-O (Stretching)	1187.94	1148.4

**Table 3:** Physicochemical parameters of prepared sublingual films.

Formulations									
Parameters	F1A	F1B	F1C	F2A	F2B	F2C	F3A	F3B	F3C
Weight (mg)	38.58 ± 2.06	53.57 ± 2.98	75.15 ± 3.39	25.67 ± 2.82	39.01 ± 1.71	60.41 ± 1.74	33.04 ± 1.91	45.32 ± 1.28	65.91 ± 2.07
Thickness (mm)	0.36 ± 0.01	0.47 ± 0.01	0.61 ± 0.02	0.29 ± 0.02	0.40 ± 0.02	0.52 ± 0.01	0.31 ± 0.02	0.43 ± 0.01	0.56 ± 0.01
Tack test	Non-tacky	Non-tacky	Non-tacky	Non-tacky	Non-tacky	Non-tacky	Non-tacky	Non-tacky	Non-tacky
Folding endurance (times)	144.66 ± 5.5	120.33 ± 4.04	92.66 ± 5.03	168.66 ± 4.50	134.33 ± 2.51	98.66 ± 5.03	173.33 ± 4.50	141.33 ± 3.05	113.33 ± 6.02
Surface pH	6.66 ± 0.05	6.43 ± 0.11	6.26 ± 0.05	6.66 ± 0.11	6.53 ± 0.11	6.33 ± 0.05	6.53 ± 0.11	6.43 ± 0.05	6.23 ± 0.05
Disintegration time (Sec)	51.33 ± 2.51	58.66 ± 2.51	66.66 ± 3.21	28.66 ± 3.05	41.33 ± 3.51	48.66 ± 2.51	35.66 ± 3.05	47.66 ± 2.08	55.33 ± 3.51
Drug content (%)	86.72 ± 2.25	77.64 ± 2.38	71.57 ± 2.76	96.79 ± 0.63	93.71 ± 0.55	90.69 ± 0.88	95.13 ± 1.05	92.46 ± 0.48	89.44 ± 0.81
% Drug release (30 Sec)	85.80 ± 1.62	77.57 ± 2.0	71.57 ± 1.84	94.24 ± 1.22	89.93 ± 3.33	85.70 ± 2.48	91.19 ± 2.20	87.46 ± 1.79	82.31 ± 1.68

**Table 4:** DSC data of nisoldipine, physical mixture, and F2A.

Sample	Sample Wt (mg)	Quantity of Drug (mg)	Melting Point	Observed	Sample
Nisoldipine	250	0.33	151.24 °C	160.82	-
Physical mixture	250	0.33	150.28 °C	17.61	11.15%
F2A	250	0.33	139.21 °C	36.71	0.21%

**Table 5:** Composition of sublingual films of nisoldipine.

Ingredients	F1A	F1B	F1C	F2A	F2B	F2C	F3A	F3B	F3C
NISSD *	20	20	20	20	20	20	20	20	20
HPC EF (mg)	200	300	400	-	-	-	-	-	-
HPMC E5 (mg)	-	-	-	200	300	400	-	-	-
HPMC E15 (mg)	-	-	-	-	-	-	200	300	400
PEG 400 (mL)	0.20	0.30	0.40	0.20	0.30	0.40	0.20	0.30	0.40
Ethanol (mL)	10	10	10	10	10	10	10	10	10

* Nisoldipine–Soluplus^®^ solid dispersion containing 10 mg of nisoldipine.

## Data Availability

All data analyzed or generated during this study are included in this article.

## Abbreviations

Nisoldipine: NIS, nisoldipine solid dispersions: NISSDs, hydroxypropyl methyl cellulose: HPMC, hydroxy ethyl cellulose: HEC EF, polyethylene glycol 400: PEG 400, micrometer: µm, weight by volume: *w*/*v*, millimeter: mm, second: sec, gram per centimeter square: g/cm^2^.
